# A Retrospective Case Report on the Longevity of a Reimplanted Closed-Apex Avulsed Canine

**DOI:** 10.1155/2020/8907904

**Published:** 2020-02-13

**Authors:** Julian Leow, Robin Amanullah

**Affiliations:** Eastman Dental Hospital, 256 Grays Inn Rd, London WC1X 8LD, UK

## Abstract

For an avulsed tooth with closed apices, it is recommended that the tooth undergoes elective root canal treatment. We however present in this case report a 73-year-old Afro-Caribbean lady, with an asymptomatic, untreated, self-replanted lower left canine which suffered an avulsion 45 years ago. The patient reported no loss of function. This case illustrates the potentially positive outcome of a replanted, non-root-treated, avulsed closed-apex tooth while highlighting the significance of patient-related factors.

## 1. Introduction

Dental avulsion is described when a tooth has been completely displaced from its socket—which severs the periodontal ligament and the neurovascular connection. It constitutes 0.5%-3% of dental trauma injuries [1]. Treatment guidelines depend on the development of the root apices (open or closed) and the amount of extra oral dry time (< or > than 60 minutes) [2]. Ideally, the tooth should be reimplanted immediately. An extraoral dry period of more than 60 minutes is associated with poor prognosis due to drying of the periodontal ligament (PDL) and pulpal cells, which are key proponents to the prognosis of the tooth. Extensive PDL necrosis is likely to cause ankylosis and in growing patients will cause infraposition of the tooth.

For an avulsed tooth with closed apices, it is recommended that if replanted, the tooth undergoes elective root canal treatment [1–5]. The tooth is normally splinted with a flexible splint for up to 2 weeks instead of a rigid splint to prevent ankylosis. Antibiotics such as doxycycline are normally prescribed. Oral hygiene and dietary advice such as soft foods for 2 weeks are usually recommended [6]. Patients are then followed up regularly. Some contraindications for replanting avulsed teeth include a patient who is immunocompromised, deciduous teeth and severe cardiac defects, unrestorable decay, severe uncontrolled diabetes, and severe mental disability. Long-standing, widely regarded guidelines have been available for dealing with dental trauma cases, and there are numerous studies on the outcome of avulsed teeth [1, 3–9]. There is however a scarcity of studies which describe the outcome of replanted closed-apex permanent teeth which have not undergone root canal treatment (RCT)—such as abscess formation, discoloration, and tooth loss. Most studies report performing root canal treatment on replanted closed-apex teeth [8]. This case report is a retrospective patient recall of the lower left canine (LL3) avulsion experienced 45 years ago, which was self-replanted into the socket and untreated.

## 2. Case Report

A 73-year-old Afro-Caribbean lady attended the dental practice in 2018 complaining of sensitivity when brushing the upper left side. She was fit and well and did not smoke or drink any alcohol. Her dental history revealed that she experienced avulsion of the LL3 due to trauma in 1973. The tooth however remained within the oral cavity and was instinctively repositioned by the patient into the socket. She attended her dentist the following day who decided against any further treatment (i.e., no RCT and no splinting). Following on from this first review of the tooth, there have been no reported symptoms relating to limited function or otherwise.

**Figure 1:**
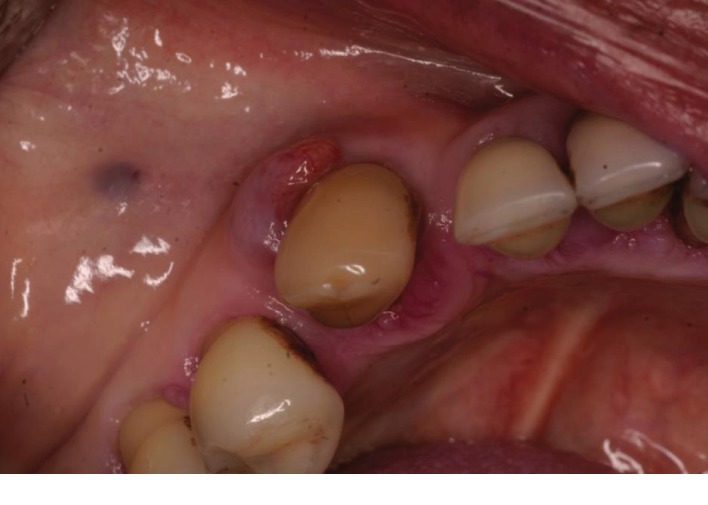
Occlusal view of LL3 before treatment.

**Figure 2:**
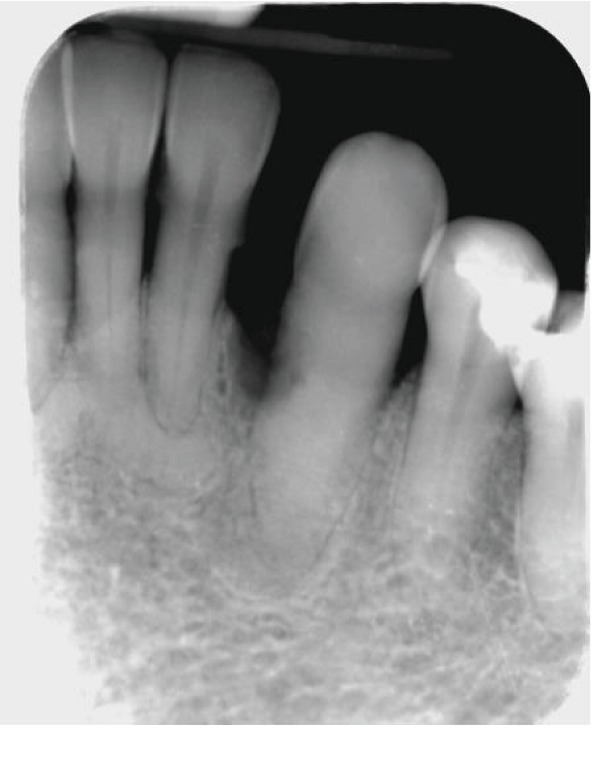
Long-cone periapical radiograph of the LL3 showing loss of about 50% bony support, obliteration of the root canal, radiolucency on the mesial surface of the root, and sclerosing osteitis periapically. No signs of caries intraorally. The LL2, LL1, and LR1 show 50-70% bone loss and bony sclerosis apically which could indicate late-stage periapical cemento-osseous dysplasia.

Both extraoral and intraoral medicine-related observations were unremarkable. After a basic periodontal examination screening, a 6-point pocket chart and appropriate radiographs were taken. The patient was diagnosed with generalised severe chronic periodontitis and UL4 cavity and was thus scheduled for nonsurgical root surface debridement and restoration of UL4. Despite the LL3 being out of the line of the arch form and displaying grade 2 mobility, the patient reported no symptoms from the tooth such as loss of function or pain. Furthermore, the LL3 was notably discolored (Figure 1) and bleeding on all sites with deep pockets. The tooth was unresponsive to the electric pulp test, which may be due to either a necrotic or obliterated pulp in the absence of periapical radiolucency. A periapical radiograph (Figure 2) of the LL3 showed reduced bony support, complete obliteration of its canal, and radiolucency involving the mesial aspect of the tooth at the midroot level. Expected outcomes of replantation of a tooth include ankylosis or external root resorption [2]. The patient was not concerned about the aesthetics.

**Figure 3:**
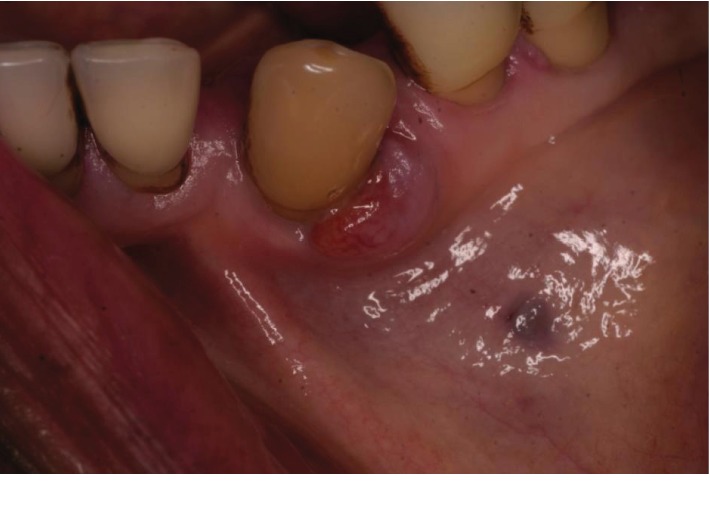
Buccal view of LL3 before treatment.

At the following appointment, the LL3 presented with an exophytic, nonpedunculated red swelling in the buccodistal gingiva which was 10 mm in diameter (Figures 1 and 3). The LL3 was unrestored and was free from coronal caries. It was not tender to vertical percussion. Due to the long history of symptomless and issueless use of the tooth, the patient stated from the start that she was opposed to have it extracted. A provisional diagnosis of lateral periodontal abscess with secondary external root resorption was made. The treatment provided was oral hygiene advice and nonsurgical root surface debridement.

Future advanced treatment options were discussed with several specialist periodontal colleagues, and the treatment proposed was open surgical access for root surface debridement and appropriate restorative intervention on the radiolucent lesion seen radiographically, with or without local periodontal regeneration.

**Figure 4:**
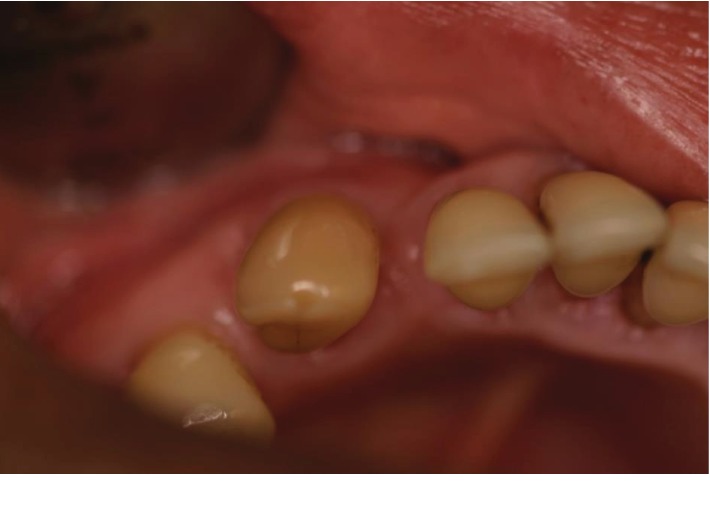
Occlusal view of LL3 after treatment.

**Figure 5:**
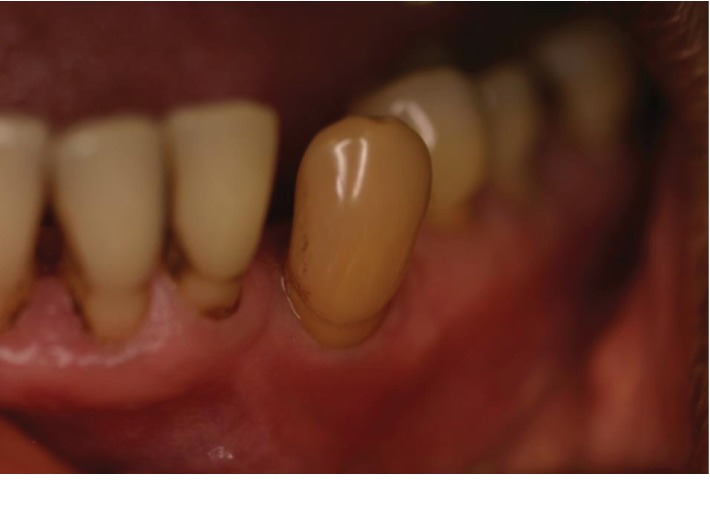
Buccal view of LL3 after treatment.

At her review appointment 2 weeks later, the swelling had resolved and she was happy with the outcome of the treatment. The patient was symptom free (Figures 4 and 5) and reported returning to eating as normal. The patient was given the option stated above; however, she decided to monitor the tooth. A 3-month recall for reassessment was planned; however, the patient never returned.

## 3. Discussion

International Association of Dental Traumatology (IADT), American Association of Endodontists (AAE), and other guidelines and studies state that root canal treatment must be commenced 7-14 days after replantation of a closed-apex tooth as “revascularisation is not expected” and inflammatory root resorption is a risk [2, 3, 7]. Further risks include abscess formation and ankylosis.

This case illustrates the positive outcome of replantation of a closed-apex tooth without further treatment. This is demonstrated via the LL3's symptom-free 45-year survival postavulsion with no reported loss of function (i.e., mastication). This is likely due to a combination of factors including nil extraoral time, nil dry time, immediate replantation [2, 9], and patient factors.

Although the outcome of this case does not meet the IADT criteria [2] for favourable outcome, it is a functional tooth which the patient was able to use for 45 years.

This case demonstrates how clinical outcomes may fall outside those “expected” as documented in the literature. For this patient, the 45 years and the ongoing function of this tooth are regarded as a positive outcome. Multifactorial reasons for this would include individual patient factors such as adaptation to less than ideal conditions of teeth, tolerance, and host response. Taking into consideration the long-term positive outcome of this tooth, its response to treatment, and this particular patient's factors, the prognosis of this tooth is deemed good. A possible reason for the absence of ankylosis is the retention of the tooth in the oral cavity after avulsion and immediate reimplantation which are favourable factors for the viability of the periodontal ligament cells.

Clearly, there is a limitation in that this highlights a single tooth-single case scenario, but a remarkable one at that. Reliance upon a patient history may carry bias, but given the clinical presentation of a 45-year-old avulsed tooth and associated symptoms, which also responded positively to the treatment, it is certainly notable in the least.

## 4. Conclusion and Clinical Implications

This case illustrates the potential long-term survivability and useful function of replanted, non-root-treated, avulsed closed-apex teeth and shows the importance of patient factors as well. A study collecting data on patients with closed-apex teeth who decline root canal treatment postavulsion would be able to further support this case report in justifying the “leave” option in select patients.
